# Baboons

**Published:** 1882-04

**Authors:** 


### BABOONS.

They resemble dogs in the general form and the length of the face or snout, but they have hands with well-developed thumbs on both the fore and hind limbs, and this, with something in the expression of the face and their habit of sitting up and using their hands in a very human fashion, at once shows that they belong to the monkey tribe. Many of them are very ugly, and in their wild state they are the fiercest and most dangerous of monkeys. Some have the tail very long, others of medium length, while it is sometimes reduced to a mere stump, and all have large cheek-pouches and bare seat-pads. They are found all over Africa, from Egypt to the Cape of Good Hope, while one species, called the hamadryas, extends from Abyssinia across the Red Sea into Arabia, and is the only baboon found out of Africa. This species was known to the ancients, and it is often represented in Egyptian sculptures, while mummies of it have been found in the catacombs. The largest and most remarkable of all the baboons is the mandrill of West Africa, whose swollen and hog-like face is ornamented with stripes of vivid blue and scarlet. This animal has a tail scarcely two inches long, while in size and strength it is not much inferior to the gorilla. These large baboons go in bands, and are said to be a match for any other animals in the African forests, and even to attack and drive away the elephants from the districts they inhabit. Turning now to Asia, we have first one of the best known of the large man-like apes—the orang-utan, found only in the two large islands Borneo and Sumatra. The name is Malay, signifying "man of the woods," and it should be pronounced órang-óotan, the accent being on the first syllable of both words. It is a very curious circumstance that, whereas the gorilla and chimpanzee are both black, like the negroes of the same country, the orang-utan is red or reddish brown, closely resembling the color of the Malays and Dyaks who live in the Bornean forests. Though very large and powerful, it is a harmless creature, feeding on fruit and never attacking any other animal except in self-defence. A full-grown male orang-utan is rather more than four feet high, but with a body as large as that of a stout man and with enormously long and powerful arms. Another group of true apes inhabit Asia and the larger Asiatic islands, and are in some respects the most remarkable of the whole family. These are the gibbons, or long-armed apes, which are generally of small size and of a gentle disposition, but possessing the most wonderful agility. In these creatures the arms are as long as the body and legs together, and are so powerful that a gibbon will hang for hours suspended from a branch, or swing to and fro and then throw itself a great distance through the air. The arms, in fact, completely take the place of the legs for travelling. Instead of jumping from bough to bough and running on the branches, like other apes and monkeys, the gibbons move along while hanging suspended in the air, stretching their arms from bough to bough, and thus going hand over hand as a very active sailor will climb along a rope. The strength of their arms is, however, so prodigious, and their hold so sure, that they often loose one hand before they have caught a bough with the other, thus seeming almost to fly through the air by a series of swinging leaps; and they travel among the network of interlacing boughs a hundred feet above the earth with as much ease and certainty as we walk or run upon level ground, and with even greater speed. These little animals scarcely ever come down to the ground of their own accord, but when obliged to do so they run along almost erect, with their long arms swinging round and round, as if trying to find some tree or other object to climb upon. They are the only apes who naturally walk without using their hands as well as their feet, but this does not make them more like men, for it is evident that the attitude is not an easy one and is only adopted because the arms are habitually used to swing by, and are therefore naturally held upward instead of downward, as they must be when walking on them.—Wallace, *in the Contemporary Review.*

